# Premenstrual Disorders, Timing of Menopause, and Severity of Vasomotor Symptoms

**DOI:** 10.1001/jamanetworkopen.2023.34545

**Published:** 2023-09-19

**Authors:** Yihui Yang, Unnur A. Valdimarsdóttir, JoAnn E. Manson, Lynnette Leidy Sievert, Bernard Leslie Harlow, A. Heather Eliassen, Elizabeth R. Bertone-Johnson, Donghao Lu

**Affiliations:** 1 Unit of Integrative Epidemiology, Institute of Environmental Medicine, Karolinska Institutet, Stockholm, Sweden; 2 Department of Epidemiology, Harvard T. H. Chan School of Public Health, Boston, Massachusetts; 3 Center of Public Health Sciences, Faculty of Medicine, University of Iceland, Reykjavik, Iceland; 4 Channing Division of Network Medicine, Department of Medicine, Brigham and Women’s Hospital, Harvard Medical School, Boston, Massachusetts; 5 Division of Preventive Medicine, Brigham and Women’s Hospital, Harvard Medical School, Boston, Massachusetts; 6 Department of Anthropology, University of Massachusetts Amherst, Amherst; 7 Department of Epidemiology, Boston University School of Public Health, Boston, Massachusetts; 8 Department of Nutrition, Harvard T. H. Chan School of Public Health, Boston, Massachusetts; 9 Department of Biostatistics and Epidemiology, School of Public Health and Health Sciences, University of Massachusetts Amherst, Amherst; 10 Department of Health Promotion and Policy, School of Public Health and Health Sciences, University of Massachusetts Amherst, Amherst

## Abstract

**Importance:**

Although premenstrual disorders (PMDs) end at menopause, it is unclear whether they are associated with the timing and symptom severity of menopause.

**Objective:**

To prospectively examine whether women with PMDs have increased risks of early menopause and menopause-related vasomotor symptoms (VMS).

**Design, Setting, and Participants:**

This population-based cohort study was nested in the Nurses’ Health Study II (data collected from questionnaire sent between June 1991 and June 2017). Analysis of menopause timing included participants who did not have natural or surgical menopause before study entry, while the analysis of VMS was restricted to women who provided information on VMS. Data were analyzed from August 2022 to March 2023.

**Exposures:**

PMDs were identified by self-reported diagnosis and confirmed with symptom questionnaires from 1991 to 2005. Participants were age-matched to women without PMD diagnoses and confirmed absence of or minimal premenstrual symptoms.

**Main Outcomes and Measures:**

During follow-up through 2017, timing of natural menopause was assessed biennially, and VMS were assessed in 2009, 2013, and 2017. The association of PMDs with early menopause was assessed by Cox proportional hazards models and with VMS by logistic regression models.

**Results:**

Of 1220 included women with PMDs, the median (IQR) age was 40.7 (37.3-43.8) years; of 2415 included women without PMDs, the median (IQR) age was 41.7 (38.3-44.8) years. The median (IQR) follow-up in this study was 20.3 (17.8-22-2) years. Early natural menopause (menopause before age 45 years) was reported by 17 women with PMDs (7.1 per 1000 person-years) and 12 women without PMDs (2.7 per 1000 person-years; adjusted hazard ratio, 2.67; 95% CI, 1.27-5.59). In addition, 795 women with PMDs (68.3%) and 1313 women without PMDs (55.3%) reported moderate or severe VMS (adjusted odds ratio, 1.68; 95% CI, 1.32-2.14). There was no observed association between PMDs and mild VMS (adjusted odds ratio, 0.99; 95% CI, 0.76-1.28).

**Conclusions and Relevance:**

In this cohort study of US women, PMDs were associated with increased risks of early menopause and moderate or severe VMS. PMDs may be indicative of underlying physiology linked to early menopause and VMS, suggesting a phenotype observable during the reproductive years that may allow clinicians to target women at risk of earlier menopause and subsequent health risks later in the life course.

## Key Points

**Question:**

Is presence of a premenstrual disorder (PMD) associated with higher risk of early menopause and menopause-related vasomotor symptoms (VMS)?

**Findings:**

In this cohort study of 3635 female participants in the US, presence of a PMD was significantly associated with higher risks of early menopause and moderate or severe VMS.

**Meaning:**

These results suggest a phenotype observable during the reproductive years that may allow clinicians to target women at risk of adverse experiences during menopause transition.

## Teaser

This cohort study examines whether US women with PMDs have increased risks of early menopause and menopause-related vasomotor symptoms.

## Introduction

Premenstrual disorders (PMDs) refer to recurrence of affective and physical symptoms before menstruation. Premenstrual syndrome (PMS) is a mild form and affects 20% to 30% women of reproductive age. Premenstrual dysphoric disorder (PMDD), though less common than PMS, is dominated by affective symptoms and can severely affect women’s quality of life.^1,2^ Although PMDs have a lasting negative impact on patients’ life,^3^ to our knowledge, long-term adverse outcomes have not been evaluated, except for increased risk of hypertension^4^ and suicidality.^5^ PMDs end at menopause, yet the biological vulnerability to hormonal fluctuations underlying PMDs may predispose individuals to adverse outcomes during the menopause transition, also characterized by dramatic fluctuations in sex hormones.

Early natural menopause and bothersome vasomotor symptoms (VMS) are challenging issues during menopause transition.^6,7,8^ PMDs, early menopause, and VMS share risk factors (eg, childhood abuse,^9,10,11^ earlier pubertal development,^12,13,14^ and smoking^15,16,17^), suggesting common etiologies. Specifically, an altered neuroendocrine system (eg, dysregulated hypothalamic-pituitary pathway) may contribute to the development of early menopause^18^ and VMS.^19^ Evidence also suggests that the interplay between the hypothalamic-pituitary-adrenal axis and the hypothalamic-pituitary-gonadal axis may be associated with PMDs.^20^ It is plausible that PMDs, or factors underlying PMDs, are associated with the development of early menopause and/or VMS.

To our knowledge, the association between PMDs and early menopause has not been studied. Furthermore, the association between PMDs and VMS remains unclear: 5 studies reported women having PMDs were more likely to endorse any or frequent VMS,^21,22,23,24,25^ whereas 1 study found a null association.^26^ However, most studies included premenopausal women, did not use validated tools to assess PMDs, and/or lacked adjustment for confounders. Here, we examined whether women with PMDs had higher risks of early natural menopause and VMS using data from a large, ongoing, and prospective cohort study from US.

## Methods

### Study Design

The Nurses’ Health Study II (NHSII) is a cohort of US female nurses. In 1989, 116 429 participants aged 25 to 42 years were enrolled and sent questionnaires regarding health behaviors and disease history.^27^ They were followed up biennially until 2017. The cumulative response rate is 88%. This study was approved by the Institutional Review Boards of Brigham and Women’s Hospital and the Swedish Ethical Review Authority. Returning of questionnaires implied informed consent. This study followed the Strengthening the Reporting of Observational Studies in Epidemiology (STROBE) reporting guideline.

We conducted a matched cohort study nested in the NHSII. The details have been described elsewhere.^4,28^ Briefly, this study included 4077 women with PMDs and 3202 without PMDs, who were sampled from women free of PMDs in 1989 and reported incident PMDs diagnoses from 1992 to 2005 (potential PMDs) or never reported PMDs (potential non-PMDs). These women were verified by a questionnaire adapted from the Calendar of Premenstrual Experiences,^29^ and all women were frequency matched on age at diagnosis/reference year. Details of study sampling are described in the eMethods in Supplement 1.

As described elsewhere,^4^ PMDs were confirmed if they satisfied 5 criteria (eTable 1 in Supplement 1). This method has been shown to be comparable with prospective symptom diaries^28^ and validated by showing a positive predictive value of 80%.^30^ Women with PMDs were classified into 2 subtypes: PMS and probable PMDD; the latter was defined using criteria established previously^31^ (eTable 1 in Supplement 1). Women were confirmed as being free from PMDs (ie, non-PMDs) if they did not report a diagnosis of PMDs on any NHSII questionnaire between 1989 and 2005 and reported either no premenstrual symptoms or mild symptoms without impact on social functioning on the PMDs assessment questionnaire.

Among individuals with self-reported PMDs (n = 4077) and non-PMDs (n = 3202), those who did not return the PMD questionnaire or did not meet criteria for PMDs or non-PMDs were excluded, leaving 1226 women with confirmed PMDs and 2417 with non-PMDs. This study further excluded women who had menopause or unknown menopause status, oophorectomy, hysterectomy, or cancer or who were lost to follow-up before study entry and did not provide information on VMS.

### Menopause Timing

On each biennial cycle, participants reported if they had permanent cessation of menstrual periods. They were provided with 4 answer options: no, yes (no periods), yes (being postmenopausal but still having periods induced by hormones), and not sure. Individuals who answered yes further provided their age at which periods ceased and the cause of menopause. Age at menopause was defined as age after 12 consecutive months of amenorrhea. Early, normal, and late menopause were defined as age at menopause younger than 45 years, 45 to 54 years, and 55 years or older, respectively.^32^ For few participants who reported being postmenopausal on 1 cycle but being premenopausal in a following cycle, age at menopause was defined as age at which menstrual periods ceased for at least 1 year, and this status was confirmed on 3 subsequent questionnaires.

### VMS

In 2009, 2013, and 2017, participants reported if they had hot flashes or night sweats (1) during the past 4 weeks and (2) at the beginning of menopause; if so, participants were asked to assess the severity as mild, moderate, or severe. VMS was defined as either report of (1) VMS in the past 4 weeks on any survey (assessed for all women) or (2) VMS at the beginning of menopause in the first cycle postmenopausal (limited to women who reported menopause during follow-up). These women were further classified as having mild VMS or moderate to severe VMS.

In addition, individuals who reported VMS at the beginning of menopause were asked about their duration of symptoms with the following options: less than 5 years, 5 to 9 years, or 10 or more years. This information was used to classify VMS duration into less than 5 years and 5 or more years.

### Covariates

We obtained information on race and ethnicity and early-life factors, including age at menarche, maternal education level, and experiences of childhood abuse, from baseline, follow-up, and supplemental questionnaires.^9^ Race and ethnicity were self-reported and categorized by investigators as non-Hispanic American Indian or Alaska Native, non-Hispanic Asian, non-Hispanic Black, Hispanic, non-Hispanic Native Hawaiian or Other Pacific Islander, and non-Hispanic White. We evaluated covariates for inclusion based on factors associated with PMDs and/or menopause in prior literature. Information on the following factors were obtained from questionnaires completed at diagnosis or reference year (1992 to 2005) or 1 to 5 years prior: marital status (married, never married, divorced, separated, or widowed), body mass index (BMI), parity (number of full-term pregnancies), total breastfeeding (in months), smoking status (never, past, or current), physical activity (in metabolic equivalent tasks hours per week), alcohol intake, and intake of vitamin D, calcium, vitamin B1, vitamin B2, iron, potassium, and zinc from food and supplements (continuous). Multiple imputation by chained equations was used to fill missing data in these covariates.^33^ In addition, we collected information on use of oral contraceptives and hormone therapy reported biennially. Information on anxiety and depression diagnoses and symptoms from 1991 to 2005 were collected from NHSII and the PMDs assessment questionnaires. More details are described in the eMethods in Supplement 1.

### Statistical Analysis

We compared background variables between women having PMDs and those free of PMDs using the Mann-Whitney *U* test and the χ^2^ test. In the analysis of menopause timing, we used multivariable Cox proportional hazards regressions to estimate hazard ratios (HRs) and 95% CIs of menopause in relation to PMDs. Attained age was used as the time scale. To account for left truncation, we specified age at entry in the Cox model.^34,35^ In addition, we split the follow-up time at age 45 years and 55 years to derive HRs of early, normal, and late menopause. To fulfill the proportional hazards assumption tested by examining the Schoenfeld residuals, we started the follow-up from age 42 years due to lack of events before that among women with PMDs or age at matching, whichever came later. We censored follow-up at onset of menopause, first report of hysterectomy, oophorectomy, cancer (except nonmelanoma skin cancer), death, loss to follow-up, or return of the 2017 questionnaire, whichever came first. Individuals who had natural or surgical menopause or cancer, who died, or who were lost to follow-up before cohort entry were excluded.

Analysis of VMS was restricted to women who provided information on VMS. We used multivariable logistic regression to estimate odds ratios (ORs) and 95% CIs of VMS among women with PMDs vs without. To provide insights into different phenotypes of VMS, we used multinomial logistic regression to analyze the association with PMDs by severity and duration of VMS.

We fitted 2 models. Model 1 was controlled for age at matching, race and ethnicity, maternal education level, and marital status. Model 2 was further adjusted for potential confounders based on prior knowledge, measured at diagnosis or reference year or 1 to 5 years prior, including category of BMI (underweight, normal weight, overweight, or obese),^36,37,38^ age at menarche (continuous),^12,13,14^ parity (0 to 1 or 2 or more children), smoking (never, past, or current),^15,16,17^ alcohol drinking (0, 0.1 to 10, or more than 10 grams per day),^39,40,41^ physical activity (continuous),^42,43,44^ childhood abuse (yes or no),^9,10,11^ vitamin D intake (by quantile), and calcium intake (by quantile).^45,46,47^

We focused on the associations with early menopause and moderate or severe VMS in the following analyses. To determine the potential differences in outcomes among PMDs by subtypes, we examined separately the associations for (1) PMS and probable PMDD and (2) PMDs with or without depression and anxiety. We also conducted several additional analyses to test risk modification and robustness of findings. Details can be found in the eMethods in Supplement 1.

All analyses were conducted in SAS version 9.4 (SAS Institute) and R version 4.1.3 (The R Foundation). A 2-tailed *P* value less than .05 was considered statistically significant. Data were analyzed from August 2022 to March 2023.

## Results

Of 1220 included women with PMDs, the median (IQR) age was 40.7 (37.3-43.8) years; of 2415 included women without PMDs, the median (IQR) age was 41.7 (38.3-44.8) years. A total of 12 women (0.3%) were American Indian or Alaska Native, 30 (0.8%) were Asian, 25 (0.7%) were Black, 54 (1.5%) were Hispanic, 2 (0.1%) were Native Hawaiian or Other Pacific Islander, and 3512 (96.6%) were White. A total of 1220 women with PMDs and 2415 matched women without were included in this study after excluding those who had menopause, cancer, or unknown menopause status or were lost to follow-up before study entry and did not provide information on VMS from confirmed PMDs (n = 1226) and non-PMDs (n = 2417). In the analysis of menopause timing, we excluded individuals who had menopause, cancer, or unknown menopause status before cohort entry, yielding a final sample of 1140 women with PMDs and 2344 women without PMDs. In the analysis of VMS, we included 1167 women with PMDs and 2379 women without PMDs who provided information on VMS (Figure).

**Figure.**
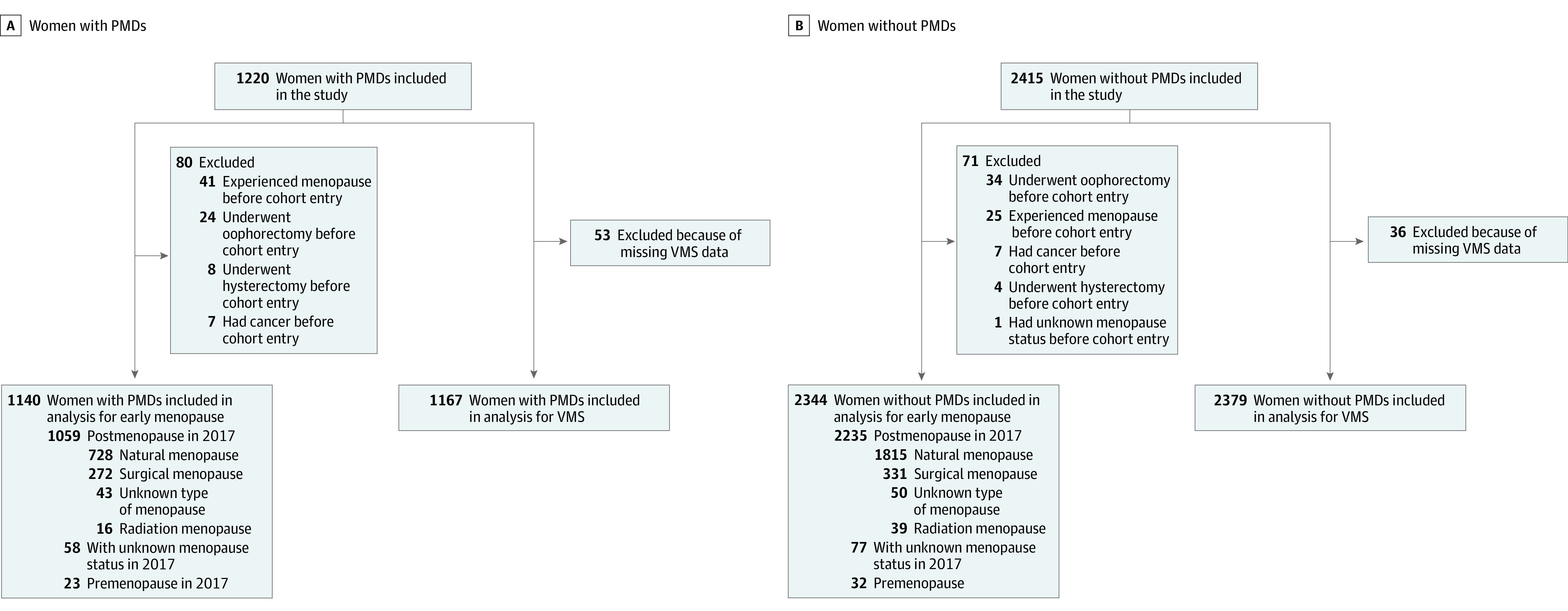
Flowchart on the Selection of Eligible Participants PMD indicates premenstrual disorder; VMS, vasomotor symptoms.

The median (IQR) follow-up was 20.3 (18.2-22.2) years in women with PMDs and 20.3 (17.1-22.2) years in women without PMDs. Compared with individuals without PMDs, those with PMDs were younger and more likely to have a lower maternal education level, become overweight or obese, smoke, use oral contraceptives, and experience childhood abuse, depression, and anxiety (Table 1).

**Table 1.** Characteristics of Women With and Without Premenstrual Disorders (PMDs) at Diagnosis or Reference Year

Characteristic	No. (%)^a^	
	Women without PMDs	Women with PMDs
Total, No.	2415	1220
Age, median (IQR), year	41.7 (38.3-44.8)	40.7 (37.3-43.8)
Year of birth		
1946-1950	200 (8.3)	110 (9.0)
1951-1955	812 (33.6)	339 (27.8)
1956-1960	1053 (43.6)	495 (40.6)
1961-1965	350 (14.5)	276 (22.6)
Race and ethnicity^b^		
American Indian or Alaska Native	7 (0.3)	5 (0.4)
Asian	22 (0.9)	8 (0.7)
Black	17 (0.7)	8 (0.7)
Hispanic	35 (1.4)	19 (1.6)
Native Hawaiian or Other Pacific Islander	1 (<0.1)	1 (0.1)
White	2333 (96.6)	1179 (96.6)
Maternal education level		
High school and below	1503 (62.2)	807 (66.1)
College and above	912 (37.8)	413 (33.9)
Marital status		
Not married	444 (18.4)	249 (20.4)
Married	1971 (81.6)	971 (79.6)
BMI^c^		
Underweight	43 (1.8)	10 (0.8)
Normal weight	1466 (60.7)	666 (54.6)
Overweight	531 (22.0)	302 (24.8)
Obese	375 (15.5)	242 (19.8)
Age at menarche, median (IQR), y	12.0 (12.0-13.0)	12.0 (11.0-13.0)
Parity		
0-1	667 (27.6)	326 (26.7)
≥2	1748 (72.4)	894 (73.3)
OC use		
No	526 (21.8)	154 (12.6)
Yes	1885 (78.1)	1065 (87.3)
Unknown	4 (0.2)	1 (0.1)
Physical activity, median (IQR), METs/wk	13.5 (5.6-27.8)	13.2 (5.3-25.8)
Smoking		
Never	1818 (75.3)	742 (60.8)
Past	449 (18.6)	357 (29.3)
Current	148 (6.1)	121 (9.9)
Alcohol drinking		
0	946 (39.2)	447 (36.6)
0.1-10 g/d	1184 (49.0)	627 (51.4)
>10 g/d	285 (11.8)	146 (12.0)
Nutrition		
Total vitamin D intake		
Quintile 1	497 (20.6)	250 (20.5)
Quintile 2	510 (21.1)	242 (19.8)
Quintile 3	509 (21.1)	248 (20.3)
Quintile 4	459 (19.0)	247 (20.2)
Quintile 5	440 (18.2)	233 (19.1)
Total calcium intake		
Quintile 1	499 (20.7)	254 (20.8)
Quintile 2	513 (21.2)	256 (21.0)
Quintile 3	479 (19.8)	237 (19.4)
Quintile 4	491 (20.3)	221 (18.1)
Quintile 5	433 (17.9)	252 (20.7)
Trauma/psychological disorders		
Childhood abuse		
No	878 (36.4)	269 (22.0)
Yes	1537 (63.6)	951 (78.0)
Depression^d^		
No	1661 (68.8)	604 (49.5)
Yes	517 (21.4)	583 (47.8)
Unknown	237 (9.8)	33 (2.7)
Anxiety^e^		
No	2026 (83.9)	1009 (82.7)
Yes	152 (6.3)	177 (14.5)
Unknown	237 (9.8)	34 (2.8)

Abbreviations: BMI, body mass index; MET, metabolic equivalent of task; OC, oral contraceptives.

^a^ Missing values in continuous variables (age at menarche [n = 13] and physical activity [n = 6]), binary variables (childhood abuse [n = 418], maternal education level [n = 136], and marital status [n = 14]), and multilevel categorical variables (smoking [n = 7], BMI category [n = 13], alcohol drinking [n = 22], and vitamin D and calcium levels [n = 22]) were imputed using predictive mean matching, binary logistic regression, and multinomial logistic regression, respectively. Missing values in OC use (n = 5), depression (n = 270), and anxiety (n = 271) were not imputed.

^b^ Data on race and ethnicity are self-reported. Due to few numbers, the American Indian or Alaska Native, Asian, Black, Hispanic, and Native Hawaiian or Other Pacific Islander race and ethnicity categories were merged into 1 category in modelling.

^c^ Calculated as weight in kilograms divided by height in meters squared. According to the extended International Obesity Task Force, underweight indicated a BMI less than 18.5; normal weight, 18.5 to 25; overweight, 25 to 30; and obese, more than 30.

^d^ Depression was defined as self-reported diagnosis, use of antidepressants, or score less than 60 on the 5-item Mental Health Inventory.

^e^ Anxiety was defined as self-reported use of minor tranquilizers or score greater than 6 on the Crown-Crisp Anxiety Scale.

### Early Menopause

A total of 1059 women (92.9%) with PMDs and 2235 women (95.3%) without PMDs reached menopause during the study period, respectively. The mean (SD) age at natural menopause was 51.4 (0.1) years and 51.8 (0.1) years among women with and without PMDs, respectively (*P* < .001), after adjusting for age at matching. Compared with women without PMDs, those with PMDs had a similar timing of natural menopause overall (adjusted HR, 0.99; 95% CI, 0.91-1.08) (Table 2). This is explained by the null associations of PMDs with normal menopause (HR, 0.99; 95% CI, 0.90-1.09) and late menopause (HR, 0.89; 95% CI, 0.70-1.12). However, women with PMDs had an increased risk of early menopause (HR, 2.67; 95% CI, 1.27-5.59).

**Table 2.** Associations of Premenstrual Disorders (PMDs) With Risks of Menopause, Overall and by Timing of Menopause

Measure	Events, No.	Person-years, No.	IR per 1000 person-years	HR (95% CI)	
				Model 1^a^	Model 2^b^
**Natural menopause**					
Women without PMDs	1767	18 728	94.3	1 [Reference]	1 [Reference]
Women with PMDs	708	8594	82.4	0.99 (0.91-1.08)	0.99 (0.91-1.08)
**By timing of menopause** ^c^					
Early menopause					
Women without PMDs	12	4454	2.7	1 [Reference]	1 [Reference]
Women with PMDs	17	2388	7.1	2.66 (1.27-5.58)	2.67 (1.27-5.59)
Normal menopause					
Women without PMDs	1465	13 945	105.1	1 [Reference]	1 [Reference]
Women with PMDs	601	6095	98.6	0.99 (0.90-1.09)	0.99 (0.90-1.09)
Late menopause					
Women without PMDs	290	330	880.1	1 [Reference]	1 [Reference]
Women with PMDs	90	111	809.9	0.89 (0.70-1.12)	0.89 (0.70-1.12)

Abbreviations: HR, hazard ratio; IR, incidence rate.

^a^ Estimates were adjusted for year of birth, race and ethnicity, maternal education level, and marital status.

^b^ Estimates were adjusted for year of birth, race and ethnicity, maternal education level, marital status, category of body mass index, age at menarche, parity, smoking, alcohol drinking, physical activity, childhood abuse, vitamin D intake, and calcium intake.

^c^ Early, normal, and late menopause was defined as age at menopause younger than 45 years, 45 to 54 years, and 55 years and older, respectively.

### VMS

Among 3546 women who had information on VMS, 3119 (88.0%) reported any VMS. A positive association was noted between PMDs and any VMS (OR, 1.43; 95% CI. 1.13-1.80) (Table 3). Significant and seemingly stronger associations were noted for moderate or severe VMS (OR, 1.68; 95% CI, 1.32-2.14) or those with symptoms lasting for at least 5 years (OR, 1.43; 95% CI, 1.01-2.02).

**Table 3.** Associations of Premenstrual Disorders (PMDs) With Vasomotor Symptoms (VMS)^a^

Measure	Women, No.	Events, No. (%)	OR (95% CI)	
			Model 1^b^	Model 2^c^
**Any VMS**				
Women without PMDs	2379	2067 (86.9)	1 [Reference]	1 [Reference]
Women with PMDs	1167	1052 (90.1)	1.42 (1.13-1.78)	1.43 (1.13-1.80)
**By severity**				
Mild VMS				
Women without PMDs	2374	749 (31.6)	1 [Reference]	1 [Reference]
Women with PMDs	1164	254 (21.8)	0.94 (0.72-1.21)	0.99 (0.76-1.28)
Moderate or severe VMS				
Women without PMDs	2374	1313 (55.3)	1 [Reference]	1 [Reference]
Women with PMDs	1164	795 (68.3)	1.70 (1.34-2.14)	1.68 (1.32-2.14)
**By duration^d^**				
Transient VMS				
Women without PMDs	1734	997 (57.5)	1 [Reference]	1 [Reference]
Women with PMDs	841	453 (53.9)	1.14 (0.83-1.59)	1.18 (0.84-1.65)
Persistent VMS				
Women without PMDs	1734	593 (34.2)	1 [Reference]	1 [Reference]
Women with PMDs	841	330 (39.2)	1.44 (1.03-2.02)	1.43 (1.01-2.02)

Abbreviation: OR, odds ratio.

^a^ Individuals without information on VMS (n = 89) were excluded in this analysis. Individuals without information on symptom severity (n = 8) were further excluded in the analysis by severity. Individuals who lacked information on VMS at the beginning of menopause (n = 462) or duration of symptoms (n = 598) were excluded in the analysis by duration.

^b^ Estimates were adjusted for age at matching, race and ethnicity, maternal education level, and marital status.

^c^ Estimates were adjusted for age at matching, race and ethnicity, maternal education level, marital status, category of body mass index, age at menarche, parity, smoking, alcohol drinking, physical activity, childhood abuse, vitamin D intake, and calcium intake.

^d^ Using data from VMS at the beginning of menopause only. Transient and persistent VMS was defined as VMS for less than 5 years and 5 or more years, respectively.

### PMD Subtypes

In analyses of PMD subtypes, statistically comparable associations with moderate or severe VMS were observed for PMDD and PMS (OR, 1.91 [95% CI, 1.19-3.05] vs 1.68 [95% CI, 1.44-1.97]) (Table 4), although the point estimate was greater for women with PMDD. A more pronounced association for early menopause was suggested among women with PMDs without depression and anxiety, while comparable associations with moderate or severe VMS were observed between women with PMDs with and without comorbid depression or anxiety.

**Table 4.** Associations of Premenstrual Disorders (PMDs) With Risks of Early Natural Menopause and Moderate to Severe Vasomotor Symptoms (VMS) by Severity and Comorbid Depression or Anxiety

Measure	Early natural menopause				Moderate or severe VMS^a^		
	Events, No.	Person-years, No.	IR per 1000 person-years	HR (95% CI)^b^	Women, No.	Events, No. (%)	OR (95% CI)^b^
Women without PMDs	12	4454	2.7	1 [Reference]	2379	1313 (55.3)	1 [Reference]
By severity							
Women with PMS	16	2214	7.2	2.71 (1.28-5.73)	1077	731 (68.1)	1.68 (1.44-1.97)
Women with PMDD	1	174	5.8	NA	90	64 (71.1)	1.91 (1.19-3.05)
By depression or anxiety^c^							
Women with PMDs without depression and anxiety	9	1114	8.1	3.00 (1.26-7.12)	531	359 (67.9)	1.67 (1.36-2.05)
Women with PMDs with depression or anxiety	6	1198	5.0	1.90 (0.71-5.06)	603	413 (68.6)	1.70 (1.40-2.08)

Abbreviations: HR, hazard ratio; IR, incidence rate; OR, odds ratio; PMDD, premenstrual dysphoric disorder; PMS, premenstrual syndrome.

^a^ Mild VMS was not considered as an outcome event.

^b^ The estimates were adjusted for birth year (for early menopause) or age at diagnosis or reference year (for VMS), race and ethnicity, maternal education level, marital status, category of body mass index, age at menarche, parity, smoking, alcohol drinking, physical activity, childhood abuse, vitamin D intake, and calcium intake at matching.

^c^ Depression was defined as self-reported diagnosis, use of antidepressants, or score less than 60 on the 5-item Mental Health Inventory. Anxiety was defined as self-reported use of minor tranquilizers or score greater than 6 on the Crown-Crisp Anxiety Scale.

### Additional Analyses

Associations of PMDs with early natural menopause and moderate or severe VMS were largely comparable across categories of age at menarche, use of OC, and BMI (eTable 2 in Supplement 1), whereas a stronger association was found with early menopause in women who ever smoked or currently smoke. Comparable results were observed (1) when further adjusting for breastfeeding and intake of vitamins and minerals; (2) when mutually adjusting for early menopause and moderate or severe VMS; (3) when restricting to participants who had complete data on covariates; and (4) when either censoring at hormone therapy or excluding women who ever used hormone therapy (eTable 3 in Supplement 1). Adjusting for potential mediators, such as time-varying BMI and smoking, minimally altered associations for early menopause (eTable 4 in Supplement 1). Using current VMS reports only or excluding women who had hysterectomy, bilateral or unilateral oophorectomy, or cancer before menopause had no material association with the results for moderate or severe VMS (eTable 5 in Supplement 1). When analyzing specific premenstrual symptoms, a more pronounced association was observed for moderate or severe VMS with premenstrual hot flashes (OR, 3.17; 95% CI, 2.11-4.74) (eTable 6 in Supplement 1).

## Discussion

To our knowledge, this is the first prospective study showing that women with clinically significant PMDs had increased risks of early natural menopause and moderate or severe VMS. Such associations were not explained by known confounders (eg, age at menarche, childhood abuse, and smoking) and psychiatric comorbidities.

### PMDs and Menopause Timing

We are not aware of any reports on PMDs and menopause timing. With validated assessment of PMDs, prospectively collected data on menopause timing, and comprehensive adjustment for confounders, our study suggests women with PMDs have an elevated risk of early natural menopause. Depression and anxiety are common in women with PMDs^48^ and have been associated with early menopause.^49,50^ We observed a significant association among PMDs without depression and anxiety, indicating such association cannot be explained by comorbid depression and anxiety.

There are potential biological explanations to our findings. First, women with PMDs may have a blunted hypothalamic-pituitary response, as suggested by one study reporting that women with PMDD and high allopregnanolone levels had blunted nocturnal cortisol levels.^51^ The change in hypothalamic-pituitary sensitivity may be associated with reduction in pituitary feedback to estrogens,^52^ contributing to early menopause. In addition, inflammatory cytokines are involved in follicle recruitment, ovulation, and follicle atresia.^53^ It is plausible that altered inflammatory profiles in women with PMDs^30,54^ are associated with more rapid ovarian follicular depletion^55^ and acceleration of menopause.^56^ These hypotheses were further supported by our findings that associations between PMDs and early menopause are stronger in smokers, who have higher inflammatory cytokine levels^57^ and an attenuated hypothalamic-pituitary pathway.^58^ However, future research (eg, by investigating the role of inflammatory markers in this link) is warranted to clarify the underlying mechanism.

### PMDs and VMS

The association between PMDs and VMS is inconclusive.^21,22,23,24,25,26^ Most studies were small, cross-sectional, and lacked adjustment for confounders. Two prospective studies have evaluated these associations, with a maximum of 6 years of follow-up.^22,24^ The observed prevalence of VMS in both studies was low (40% to 44%), suggesting that many participants might have not entered perimenopause by the end of follow-up. Many studies used unvalidated tools to assess PMDs and assessed PMDs concurrently with VMS, which makes distinguishing both conditions difficult.

Our study used a validated assessment of PMDs and prospectively collected data over 26 years, during which more than 90% of participants have reached menopause. Our data showed increased odds of moderate or severe VMS in women with PMDs (OR, 1.68; 95% CI, 1.32-2.14). Given the prevalence of moderate or severe VMS (2108 of 3538 [59.6%]), the OR is equivalent to a relative risk of 1.20 (95% CI, 1.11-1.28).^59^ Together with seemingly stronger associations with moderate or severe VMS and persistent VMS (duration of more than 5 years) as well as between probable PMDD and VMS, this study supports a potentially important association between PMDs and VMS.

It is not surprising that women who are hypersensitive to hormone fluctuations during the menstrual cycle are also vulnerable to hormone changes around menopause. Notably, premenstrual hot flashes are the strongest correlator of menopause-related VMS, suggesting PMDs, or premenstrual hot flashes, and VMS are similar phenotypes but happening in different life stages. However, the underlying mechanism remains unknown. We propose several pathways. Similar to early menopause, the association between PMDs and VMS could be attributed to dysregulation of the hypothalamic-pituitary pathway.^60^ However, we observed a significant association between PMDs and moderate or severe VMS after controlling for early menopause, suggesting different underlying pathways between PMDs and VMS. For example, evidence suggests that women with PMDs have reduced vascular tone and reactivity (eg, higher arterial stiffness^61^), which may disrupt heat dissipation responses and present as VMS.^19^ Relatedly, in a cross-sectional study of young women in the US, a large difference in blood pressure was found by presence or absence of premenstrual hot flashes or night sweats.^62^ This suggested that PMDs and VMS may characterize a group of women identifiable during the reproductive stage who may be at higher risk of cardiometabolic conditions in later life.

### Strengths and Limitations

With prospective measurements on menopause status, timing, and VMS and detailed information on confounders, to our knowledge, our study provides the first comprehensive assessment of associations for PMDs with early menopause and moderate or severe VMS during over 2 decades of follow-up. However, several limitations should be considered. First, we did not use prospective symptom recording to assess PMDs, which is not feasible in large epidemiological studies. Alternatively, we used self-reported incident diagnosis followed by an assessment, which has been validated to have a high positive predictive value in classification of PMDs.^30^ However, as the PMDs assessment questionnaire did not align perfectly with the *Diagnostic and Statistical Manual of Mental Disorders* (Fifth Edition), there could be misclassifications in PMDD. Second, despite prospective assessment of menopause timing and type, self-report may lead to some misclassification. However, self-assessment of menopause was validated, showing a high consistency in reporting menopause status and type (98.8% agreement) and age (82% to 95% agreement).^63^ Third, perception of VMS is subjective, and we relied on self-assessed VMS. However, the prevalence of VMS in women without PMDs is comparable with that in other studies in the US.^64,65^ Although there could be additional misclassifications when participants had a history of psychological symptoms,^66^ we observed similar associations across categories of depression and anxiety. Fifth, statistical power was low for early menopause in some analyses; particularly, we lacked events before age 42 years among women with PMDs, and chance may have contributed to the findings. Sixth, the study population is homogeneous in terms of occupation, race, and ethnicity, and our results may only be generalized to White individuals. A study has shown that the association between premenstrual symptoms and VMS was weakest in White individuals.^22^ Our result of VMS might have been underestimated compared with those seen in a more racially and ethnically diverse population.

## Conclusions

In conclusion, this study suggests that women with PMDs are at increased risks of early menopause and moderate or severe VMS. PMDs may be indicative of underlying physiology linked to early menopause and VMS, suggesting a phenotype observable during the reproductive years that may allow clinicians to target women at risk of adverse experiences during menopause transition. Together with the documented links between PMDs, early menopause and VMS, and hypertension and cardiometabolic diseases, future research on assessing health risks after menopause is warranted for this group even though PMDs end at menopause.

## Notes

Supplement 1.
eMethods.
**eTable 1.** Classification Criteria of Premenstrual Disorders and Premenstrual Dysphoric Disorder **eTable 2.** Associations of Premenstrual Disorders With Risks of Early Natural Menopause and Moderate/Severe Vasomotor Symptoms in Stratified Analyses **eTable 3.** Associations of Premenstrual Disorders With Risks of Early Natural Menopause and Moderate/Severe Vasomotor Symptoms in Additional Analyses **eTable 4.** Associations of Premenstrual Disorders With Risks of Early Natural Menopause in Additional Analyses **eTable 5.** Associations of Premenstrual Disorders With Risks of Moderate/Severe Vasomotor Symptoms in Additional Analyses **eTable 6.** Associations of Specific Premenstrual Symptoms With Risks of Moderate/Severe Vasomotor Symptoms
eReferences.

Supplement 2.
Data Sharing Statement
