# Supplementary material for: Premenstrual Disorders, Timing of Menopause, and Severity of Vasomotor Symptoms
Source: JAMA Netw Open. 2023 Sep 19;6(9):e2334545. doi: 10.1001/jamanetworkopen.2023.34545 (PMC10509727; doi:10.1001/jamanetworkopen.2023.34545)
Supplement: Supplement 1. — eMethods. eTable 1. Classification Criteria of Premenstrual Disorders and Premenstrual Dysphoric Disorder eTable 2. Associations of Premenstrual Disorders With Risks of Early Natural Menopause and Moderate/Severe Vasomotor Symptoms in Stratified Analyses eTable 3. Associations of Premenstrual Disorders With Risks of Early Natural Menopause and Moderate/Severe Vasomotor Symptoms in Additional Analyses eTable 4. Associations of Premenstrual Disorders With Risks of Early Natural Menopause in Additional Analyses eTable 5. Associations of Premenstrual Disorders With Risks of Moderate/Severe Vasomotor Symptoms in Additional Analyses eTable 6. Associations of Specific Premenstrual Symptoms With Risks of Moderate/Severe Vasomotor Symptoms eReferences. [file jamanetwopen-e2334545-s001.pdf]

Supplemental Online Content

Yang Y, Valdimarsdóttir UA, Manson JE, et al. Premenstrual disorders, timing of menopause, and severity of vasomotor symptoms. *JAMA Netw Open*. 2023;6(9):e2334545. doi:10.1001/jamanetworkopen.2023.34545

eMethods.
eTable 1. Classification Criteria of Premenstrual Disorders and Premenstrual Dysphoric Disorder
eTable 2. Associations of Premenstrual Disorders With Risks of Early Natural Menopause and Moderate/Severe Vasomotor Symptoms in Stratified Analyses
eTable 3. Associations of Premenstrual Disorders With Risks of Early Natural Menopause and Moderate/Severe Vasomotor Symptoms in Additional Analyses
eTable 4. Associations of Premenstrual Disorders With Risks of Early Natural Menopause in Additional Analyses
eTable 5. Associations of Premenstrual Disorders With Risks of Moderate/Severe Vasomotor Symptoms in Additional Analyses
eTable 6. Associations of Specific Premenstrual Symptoms With Risks of Moderate/Severe Vasomotor Symptoms
eReferences.

This supplementary material has been provided by the authors to give readers additional information about their work.

eMethods.

Study design

Among women who were free of PMDs in 1989 and 1991, self-reported diagnoses between 1993 and 2005 were considered as potential PMDs. Among women who never reported PMDs during this period, each was randomly assigned a reference year comparable to diagnosis year of potential PMDs. After excluding conditions (e.g., menopause) that might lead to misclassifications, 4,077 potential PMDs and 3,202 non-PMDs were frequency matched to PMDs on age at diagnosis/reference year.

Potential PMDs and non-PMDs were mailed questionnaires based on the Calendar of Premenstrual Experiences[1], assessing occurrence of 26 premenstrual symptoms, severity of overall symptom and impact on social functioning. Participants were also asked if they had depression diagnosis and used anti-depressants, and if so, the timing. The completed questionnaire was returned by 3,548 (87%) PMDs and 3,041 (95%) non-PMDs, respectively. A total of 1,226 (35%) women with PMDs and 2,417 (79%) women without PMDs were confirmed. Individuals who did not met criteria for either PMDs or non-PMDs were excluded. The present study further excluded PMDs (n=6) and non-PMDs (n=2) who had menopause or unknown menopause status, oophorectomy, hysterectomy, cancer or loss-to-follow-up before study entry and did not provide information on VMS. Therefore, we included 1,220 women with PMDs and 2,415 women without PMDs in this study.

Covariates

Race/ethnicity and age at menarche were collected in 1989. Maternal education level was surveyed in 2005 in NHSII and 2001 in maternal questionnaires.

Factors associated with both PMDs and menopause timing/symptoms were considered as potential confounders. Experiences of abuse before age 11, including physical, sexual and emotional abuse, were collected from a violence questionnaire in 2001[2]. Information on the following covariates were obtained at diagnosis/reference year (1992-2005) or 1 year prior. Marital status, parity, smoking, and physical activity were measured every 2-4 years since 1991. Body mass index (BMI, kg/m^2) was computed using self-reported height in 1989 and weight at diagnosis/reference year, and categorized into underweight, normal, overweight, and obese based on the extended International Obesity Task Force[3]. Dietary factors, including alcohol intake and amount of vitamin D and calcium from food and supplements were collected every 4 years since 1991 using a semiquantitative food frequency questionnaire (SFFQ). Nutrition intakes were further categorized based on quintiles.

In addition, we collected information on factors only related to PMDs or menopause timing/symptoms. Total breastfeeding months was surveyed from 1993. Intake of vitamin B1, vitamin B2, iron, zinc and potassium were surveyed in the SFFQ every 4 years since 1991.

Multiple imputation with chained equations was used to impute missing values in covariates[4]. Missing values were generated using predictive mean matching (for age at menarche and physical activity), logistic (for childhood abuse, maternal education level, marital status, breast feeding), and multinomial logistic regression (for smoking, BMI category, and intake of alcohol, calcium, vitamins, and minerals) using the variables menopause status, PMDs, age at diagnosis/reference year, birth year, race, and parity.

Use of oral contraceptive and hormone therapy may delay or mask onset of menopause[5] and alleviate VMS[6]. They were surveyed biennially from 1991.

Anxiety and depression are common comorbidities of PMDs[7]. We defined women with anxiety as having a Crown-Crisp Anxiety Scale items (CCI) > 6 in 1993 and 2005[8], or ever use of minor tranquilizers surveyed in 1993 and every two years since 1997. Depression was identified by: 1) clinician-diagnosed depression, reported in NHSII questionnaire in 2003 and 2005, and PMDs assessment questionnaire; 2) antidepressant use, collected in 1993 and biennially from 1997 in NHSII, and PMDs assessment questionnaire; 3) the 5-itm Mental Health Index (MHI) scored < 60[9], assessed in 1993, 1997 and 2001 in NHS II.

Statistical analyses

We also conducted several additional analyses. First, to test potential risk modifications, we performed stratified analyses by age at menarche, use of OC, BMI category, and smoking status at matching. Second, to address residual confounding, we further adjusted for factors related with PMDs/menopause only, including breastfeeding, and intake of vitamin B1, vitamin B2, iron, zinc, and potassium at matching. Third, to evaluate the independent role of early menopause and moderate/severe VMS, we mutually adjusted for these factors.

© 2023 Yang Y et al. *JAMA Network Open*.

Fourth, to evaluate the impact of imputation, we conducted a complete case analysis. Fifth, to evaluate the impact of HT use, we censored at HT use in the analysis for early menopause and excluded women who had ever used HT in the analysis for VMS.

In the analysis of early menopause, presuming that PMD diagnosis may lead to changes in lifestyle (e.g. smoking, alcohol drinking and physical activity), BMI, and marital status, we adjusted for these factors in a time-varying manner. In the analysis of moderate/severe VMS, first we only used data from current VMS and performed mixed effects logistic regression to account for correlations between repeated measurements[10]. Moreover, we excluded women who had cancer, hysterectomy, oophorectomy before menopause to rule out potential mediation to VMS. We also evaluated the association between VMS and specific premenstrual symptoms.

© 2023 Yang Y et al. *JAMA Network Open*.

eTable 1. Classification Criteria of Premenstrual Disorders and Premenstrual Dysphoric Disorder

Participants were identified as having premenstrual disorders if they reported:	
1	at least 1 affective and 1 physical/behavioral manifestation
2	moderate/severe overall symptoms, or moderate/severe social impairment
3	symptoms starting within 2 weeks before menstruation
4	symptoms ending within 4 days after menses onset
5	symptoms absent within one week after menses end
Participants were identified as having premenstrual dysphoric disorder if they reported:	
1	at least one of four emotional symptoms: anxiety, irritability/anger, mood swings/tearful and depression
2	at least one of the following symptoms additionally occurs, and add up to at least 5 symptoms: desire for loneliness, confusion, fatigue, food craving/change in appetite, insomnia, and/or other physical symptoms
3	severe social impairment

.

© 2023 Yang Y et al. *JAMA Network Open*.

eTable 2. Associations of Premenstrual Disorders With Risks of Early Natural Menopause and Moderate/Severe Vasomotor Symptoms in Stratified Analyses

	Early natural menopause [a]				Moderate/Severe VMS [b]			
	No PMDs N (IR)	PMDs N (IR)	HR (95% CI) [c]	P for interaction	No PMDs N (%)	PMDs N (%)	OR (95% CI) [c]	P for interaction
By age at menarche								
<=12	5 (2.2)	11 (8.8)	2.54 (1.21-5.35)	0.59	692 (56.0)	440 (70.2)	1.79 (1.45-2.20)	0.46
> 12	7 (3.2)	6 (5.3)	2.67 (1.27-5.63)		621 (54.5)	355 (66.1)	1.59 (1.28-1.98)	
By OC use								
No	4 (3.9)	3 (10.1)	2.62 (1.22-5.64)	1.00	259 (50.0)	88 (60.7)	1.50 (1.03-2.20)	0.58
Yes	8 (2.3)	14 (6.7)	2.62 (1.25-5.50)		1,053 (56.9)	706 (69.4)	1.69 (1.43-1.99)	
By BMI category [d]								
Underweight	/	/	/	/	22 (51.2)	7 (70.0)	2.02 (0.46-9.00)	0.83
Normal	8 (2.8)	12 (8.4)	2.52 (1.20-5.30)	0.33	832 (57.8)	443 (69.8)	1.62 (1.33-1.99)	
Overweight/Obese	4 (2.6)	5 (5.3)	2.76 (1.31-5.83)		459 (51.5)	345 (66.5)	1.77 (1.41-2.23)	
By smoking								
No	11 (3.2)	8 (5.3)	2.40 (1.14-5.04)	0.02	955 (53.1)	466 (65.7)	1.72 (1.43-2.06)	0.78
Yes	1 (1.0)	9 (10.1)	3.02 (1.43-6.39)		358 (62.0)	329 (72.3)	1.64 (1.25-2.14)	

BMI, body mass index; CI, confidence interval; IR, incidence rate per 1,000 person-years; N, number of events; HR, hazard ratio; OR, odds ratio; PMD, premenstrual disorder; OC, oral contraceptives; PYs, person years; Ref., reference; VMS, vasomotor symptoms.

OC use, BMI category and smoking were collected at diagnosis/reference year.

[a] The follow-up started from age 42, due to no events before age 42 among women with PMDs, or age at matching, whichever came later.

[b] Mild VMS was not considered as an outcome event.

[c] The estimates were adjusted for or birth year (for early menopause)/age at diagnosis or reference year (for VMS), race, maternal education level, marital status, category of body mass index, age at menarche, parity, smoking, alcohol drinking, physical activity, childhood abuse, vitamin D intake and calcium intake at baseline.

[d] There was no case of early menopause in the category of underweight.

© 2023 Yang Y et al. *JAMA Network Open*.

eTable 3. Associations of Premenstrual Disorders With Risks of Early Natural Menopause and Moderate/Severe Vasomotor Symptoms in Additional Analyses

	Early Natural Menopause [a]			Moderate/Severe VMS [b]		
	PYs	N (IR)	HR (95%CI) [c]	Women	N (%)	OR (95% CI) [c]
Additional adjustment [d]						
No PMDs	4,454	12 (2.7)	Ref.	2,374	1,313 (55.3)	Ref.
PMDs	2,388	17 (7.1)	2.64 (1.26-5.53)	1,164	795 (68.3)	1.71 (1.46-2.00)
Mutual adjustments						
No PMDs	4,454	12 (2.7)	Ref.	2,374	1,313 (55.3)	Ref.
PMDs	2,388	17 (7.1)	2.56 (1.21-5.42)	1,164	795 (68.3)	1.70 (1.45-1.98)
Complete-case analysis						
No PMDs	3,986	11 (2.8)	Ref.	2,138	1,179 (55.1)	Ref.
PMDs	1,818	13 (7.2)	2.62 (1.17-5.84)	905	616 (68.1)	1.68 (1.41-1.99)
Censor at HT/Exclude women who used HT						
No PMDs	2,876	7 (2.4)	Ref.	1,013	514 (50.7)	Ref.
PMDs	1,362	11 (8.1)	3.30 (1.28-8.52)	288	183 (63.5)	1.55 (1.17-2.06)

CI, confidence interval; IR, incidence rate per 1,000 person-years; N, number; HR, hazard ratio; OR, odds ratio; PMD, premenstrual disorder; PYs, person years; Ref., reference.
Individuals who lacked information on any covariates (n=548 (15.1%)) were excluded.
[a] The follow-up started from age 42, due to no events before age 42 among women with PMDs, or age at matching, whichever came later, except for analysis censored at HT use, in which the follow-up started from age 43, due to no events before age 43 among women with PMDs, or age at matching, whichever came later.
[b] Mild VMS was not considered as an outcome event.
[c] The estimates were adjusted for or birth year (for early menopause)/age at diagnosis or reference year (for VMS), race, maternal education level, marital status, category of body mass index, age at menarche, parity, smoking, alcohol drinking, physical activity, childhood abuse, vitamin D intake and calcium intake at matching.
[d] The estimates were additionally adjusted for breastfeeding (ie, 0-24 months, 24 months+), intake of vitamin B1, B2, zinc, iron and potassium (in quintiles).

© 2023 Yang Y et al. *JAMA Network Open*.

eTable 4. Associations of Premenstrual Disorders With Risks of Early Natural Menopause in Additional Analyses

	PYs	N (IR)	HR (95% CI)
Adjust for potential mediators [a]			
No PMDs	4,454	12 (2.7)	Ref.
PMDs	2,388	17 (7.1)	2.72 (1.30-5.69)

CI, confidence interval; IR, incidence rate per 1,000 person-years; N, number; HR, hazard ratio; PMD, premenstrual disorder; PYs, person years; Ref., reference.

The follow-up started from age 42, due to no events before age 42 among women with PMDs, or age at matching, whichever came later.

[a] The estimates were adjusted for year of birth, race, maternal education level, age at menarche, parity, childhood abuse, vitamin D intake and calcium intake at matching, and time-varying marital status, body mass index category, smoking, alcohol drinking, and physical activity.

© 2023 Yang Y et al. *JAMA Network Open*.

eTable 5. Associations of Premenstrual Disorders With Risks of Moderate/Severe Vasomotor Symptoms in Additional Analyses

Using only recent reports to define moderate/severe VMS [a]	Reports	VMS, N (%)	OR (95% CI) [b]
No PMDs	6,677	1,204 (18.0)	Ref.
PMDs	3,077	802 (26.1)	1.57 (1.34-1.83)
Excluding individuals who had hysterectomy, oophorectomy, or cancer before menopause	Women	VMS, N (%)	OR (95% CI) [b]
No PMDs	2,144	1,185 (55.3)	Ref.
PMDs	1,012	699 (69.1)	1.73 (1.47-2.04)

CI, confidence interval; N, number; OR, odds ratio; PMD, premenstrual disorder; Ref., reference; VMS, vasomotor symptoms.
Mild VMS was not considered as an outcome event.
[a] Mixed effects logistic regression was used.
[b] The estimates were adjusted for or age, race, maternal education level, marital status, category of body mass index, age at menarche, parity, smoking, alcohol drinking, physical activity, childhood abuse, vitamin D intake and calcium intake at diagnosis/reference year.

© 2023 Yang Y et al. *JAMA Network Open*.

eTable 6. Associations of Specific Premenstrual Symptoms With Risks of Moderate/Severe Vasomotor Symptoms

	Physical symptoms				Affective/behavioral symptoms		
	Women	Moderate/severe VMS, N (%)	OR (95% CI)[a]		Women	Moderate/severe VMS, N (%)	OR (95% CI)[a]
Hot flashes				Confusion			
No	3,370	1,970 (58.5)	Ref.	No	3,406	2,010 (59.0)	Ref.
Yes	168	138 (82.1)	3.17 (2.11-4.74)	Yes	132	98 (74.2)	1.92 (1.29-2.87)
Breast tenderness				Depression			
No	1,366	733 (53.7)	Ref.	No	2,888	1,653 (57.2)	Ref.
Yes	2,172	1,375 (63.3)	1.46 (1.27-1.68)	Yes	650	455 (70.0)	1.71 (1.41-2.06)
Acne				Tendency to cry easily			
No	2,643	1,521 (57.5)	Ref.	No	2,453	1,373 (56.0)	Ref.
Yes	895	587 (65.6)	1.41 (1.20-1.66)	Yes	1,085	735 (67.7)	1.65 (1.42-1.93)
Palpitations				Desire to be alone			
No	3,394	2,011 (59.3)	Ref.	No	2,980	1,721 (57.8)	Ref.
Yes	144	97 (67.4)	1.38 (0.96-1.98)	Yes	558	387 (69.4)	1.64 (1.34-2.00)
Dizziness				Insomnia			
No	3,472	2,064 (59.4)	Ref.	No	3,138	1,831 (58.3)	Ref.
Yes	66	44 (66.7)	1.37 (0.81-2.32)	Yes	400	277 (69.2)	1.62 (1.29-2.03)
Diarrhea or constipation				Anxiety			
No	2,653	1,534 (57.8)	Ref.	No	3,053	1,770 (58.0)	Ref.
Yes	885	574 (64.9)	1.34 (1.14-1.57)	Yes	485	338 (69.7)	1.60 (1.30-1.98)
Abdominal bloating				Hypersensitivity			
No	1,900	1,064 (56.0)	Ref.	No	2,928	1,687 (57.6)	Ref.
Yes	1,638	1,044 (63.7)	1.32 (1.15-1.51)	Yes	610	421 (69.0)	1.59 (1.32-1.93)*
Lower back pain				Forgetfulness			
No	2,672	1,554 (58.2)	Ref.	No	3,239	1,901 (58.7)	Ref.
Yes	866	554 (64.0)	1.24 (1.06-1.46)	Yes	299	207 (69.2)	1.57 (1.21-2.03)
Headache				Irritability			
No	2,555	1,490 (58.3)	Ref.	No	1,613	880 (54.6)	Ref.
Yes	983	618 (62.9)	1.21 (1.04-1.41)	Yes	1,925	1,228 (63.8)	1.43 (1.24-1.64)
Swelling in the extremities				Mood swings			
No	3,079	1,822 (59.2)	Ref.	No	2,355	1,339 (56.9)	Ref.
Yes	459	286 (62.3)	1.16 (0.94-1.43)	Yes	1,183	769 (65.0)	1.38 (1.19-1.60)
Abdominal cramping				Angry outbursts			
No	2,751	1,621 (58.9)	Ref.	No	2,581	1,480 (57.3)	Ref.
Yes	787	487 (61.9)	1.13 (0.96-1.33)	Yes	957	628 (65.6)	1.37 (1.16-1.60)

© 2023 Yang Y et al. *JAMA Network Open*.

Nausea				Fatigue			
No	3,451	2,055 (59.5)	Ref.	No	2,438	1,395 (57.2)	Ref.
Yes	87	53 (60.9)	1.08 (0.70-1.69)	Yes	1,100	713 (64.8)	1.35 (1.16-1.57)
				Change in appetite			
				No	2,208	1,264 (57.2)	Ref.
				Yes	1,330	844 (63.5)	1.27 (1.10-1.46)
				Food craving			
				No	1,846	1,052 (57.0)	Ref.
				Yes	1,692	1,056 (62.4)	1.24 (1.08-1.42)

CI, confidence interval; N, number; OR, odds ratio; PMD, premenstrual disorder; Ref., reference; VMS, vasomotor symptoms.
Mild VMS was not considered as an outcome event.
[a] The estimates were adjusted for or age, race, maternal education level, marital status, category of body mass index, age at menarche, parity, smoking, alcohol drinking, physical activity, childhood abuse, vitamin D intake and calcium intake at diagnosis/reference year.

© 2023 Yang Y et al. *JAMA Network Open*.

eReferences.

1. Mortola JF, Girton L, Beck L, Yen SS. Diagnosis of premenstrual syndrome by a simple, prospective, and reliable instrument: the calendar of premenstrual experiences. *Obstet Gynecol*. 1990;76(2):302-307.

2. Bertone-Johnson ER, Whitcomb BW, Missmer SA, Manson JE, Hankinson SE, Rich-Edwards JW. Early life emotional, physical, and sexual abuse and the development of premenstrual syndrome: a longitudinal study. *J Womens Health (Larchmt)*. 2014;23(9):729-739. doi:10.1089/jwh.2013.4674

3. Cole TJ, Lobstein T. Extended international (IOTF) body mass index cut-offs for thinness, overweight and obesity. *Pediatr Obes*. 2012;7(4):284-294. doi:10.1111/j.2047-6310.2012.00064.x

4. White IR, Royston P, Wood AM. Multiple imputation using chained equations: Issues and guidance for practice. *Stat Med*. 2011;30(4):377-399. doi:10.1002/sim.4067

5. Roman Lay AA, do Nascimento CF, Horta BL, Dias Porto Chiavegatto Filho A. Reproductive factors and age at natural menopause: A systematic review and meta-analysis. *Maturitas*. 2020;131:57-64. doi:10.1016/j.maturitas.2019.10.012

6. Kaunitz AM. Oral contraceptive use in perimenopause. *Am J Obstet Gynecol*. 2001;185(2 Suppl):S32-37. doi:10.1067/mob.2001.116525

7. Kim DR, Gyulai L, Freeman EW, Morrison MF, Baldassano C, Dubé B. Premenstrual dysphoric disorder and psychiatric co-morbidity. *Arch Womens Ment Health*. 2004;7(1):37-47. doi:10.1007/s00737-003-0027-3

8. Crown S, Crisp AH. A short clinical diagnostic self-rating scale for psychoneurotic patients. The Middlesex Hospital Questionnaire (M.H.Q.). *Br J Psychiatry*. 1966;112(490):917-923. doi:10.1192/bjp.112.490.917

9. Roberts AL, Kubzansky LD, Malspeis S, Feldman CH, Costenbader KH. Association of Depression With Risk of Incident Systemic Lupus Erythematosus in Women Assessed Across 2 Decades. *JAMA Psychiatry*. 2018;75(12):1225-1233. doi:10.1001/jamapsychiatry.2018.2462

10. Diggle P, Heagerty P, Liang KY, Zeger S. *Analysis of Longitudinal Data*. 2nd ed. Oxford University Press Inc; 2002.

© 2023 Yang Y et al. *JAMA Network Open*.
