# Supplementary material for: Premenstrual Disorders, Timing of Menopause, and Severity of Vasomotor Symptoms
Source: JAMA Netw Open. 2023 Sep 19;6(9):e2334545. doi: 10.1001/jamanetworkopen.2023.34545 (PMC10509727; doi:10.1001/jamanetworkopen.2023.34545)
Supplement: Supplement 2. — Data Sharing Statement [file jamanetwopen-e2334545-s002.pdf]

Data Sharing Statement

Yang. Premenstrual Disorders, Timing of Menopause, and Severity of Vasomotor Symptoms. *JAMA Netw Open*. Published September 19, 2023. doi:10.1001/jamanetworkopen.2023.34545

Data
Data available: No

Additional Information
Explanation for why data not available: Because of patient privacy, the dataset used in this study is not publicly available.
